# A Conjugate Gradient Algorithm with Function Value Information and N-Step Quadratic Convergence for Unconstrained Optimization

**DOI:** 10.1371/journal.pone.0137166

**Published:** 2015-09-18

**Authors:** Xiangrong Li, Xupei Zhao, Xiabin Duan, Xiaoliang Wang

**Affiliations:** 1 Guangxi Colleges and Universities Key Laboratory of Mathematics and Its Applications, College of Mathematics and Information Science, Guangxi University, Nanning, Guangxi, P.R. China; 2 Mathematics and Physics Institute of Henan University of Urban Construction, Pingdingshan, Henan, P. R. China; Leibniz Institute for Age Research, GERMANY

## Abstract

It is generally acknowledged that the conjugate gradient (CG) method achieves global convergence—with at most a linear convergence rate—because CG formulas are generated by linear approximations of the objective functions. The quadratically convergent results are very limited. We introduce a new PRP method in which the restart strategy is also used. Moreover, the method we developed includes not only n-step quadratic convergence but also both the function value information and gradient value information. In this paper, we will show that the new PRP method (with either the Armijo line search or the Wolfe line search) is both linearly and quadratically convergent. The numerical experiments demonstrate that the new PRP algorithm is competitive with the normal CG method.

## Introduction

Consider

$$\begin{array}{c} \min_{x\in {ℜ}^{n}}f\left(x\right), \end{array}$$ (1)

where *f*(*x*) : ℜ^*n*^ → ℜ is a continuously differentiable function. This optimization problem can be used to model other problems (see [1–5]). The iterative processes of the conjugate gradient (CG) methods are determined by

$$\begin{array}{c} x_{k+1}=x_{k}+\alpha_{k}d_{k},\;\;k=0,\;1,\;2,\cdots , \end{array}$$ (2)

where *x*_*k*_ is the *k*-th iteration; *α*_*k*_ is the step length, which is determined by a line search method; and *d*_*k*_ is the search direction defined by

$$\begin{array}{c} d_{k}=\{\begin{array}{cc} -g_{k}, & \text{if}\;\;\;\;k=0,\\ -g_{k}+\beta_{k}d_{k-1}, & \text{if}\;\;\;\;k\geq 1, \end{array} \end{array}$$ (3)

where *g*_*k*_ = *g*(*x*_*k*_) is the gradient ∇*f*(*x*_*k*_) of *f*(*x*) at the point *x*_*k*_ and *β*_*k*_ is a scalar. In addition, the parameter *β*_*k*_ varies between different CG formulas (see [6–17]etc.). This paper focuses on the PRP method designed by [13, 14]

$$\begin{array}{c} \beta_{k}^{PRP}=\frac{g_{k}^{T}\left(g_{k}-g_{k-1}\right)}{{∥g_{k-1}∥}^{2}}, \end{array}$$ (4)

where *g*_*k*−1_ is the gradient ∇*f*(*x*_*k*−1_) of *f*(*x*) at the point *x*_*k*−1_, and ‖.‖ denotes the Euclidean norm of vectors. Recently, there have been several successful research projects involving the PRP algorithm (see [7, 18–29] for details). The linear convergence of the standard PRP method is well known. If a restart strategy is used, the algorithm can be n-step quadratic convergent [30–32]; however, the quadratically convergent results are very limited. Zhang et al. [33] presented a three-term CG algorithm by modifying the PRP (MPRP) method and proved its global convergence. Moreover, Li and Tian [34] proved that the MPRP method with a restart strategy exhibited quadratic convergence under certain inexact line searches and suitable assumptions. In this paper, we focus primarily on the MPRP method. On the basis of the MPRP method [33], a new three-term CG formula is proposed in which the formula includes not only the gradient information but also the function information. By applying the conclusion of [34], we prove that the new CG method obtains global convergence for general functions and n-step quadratic convergence for uniformly convex functions. The numerical results show that the new method performs quite well. The main attributes of this method are stated below.

- The new PRP CG method possesses not only the gradient information but also information about function values.
- The sufficient descent property is satisfied without any conditions.
- The global convergence, linear convergent rate, and n-step quadratic convergence are established.
- The numerical results demonstrate that this method is competitive with the normal CG method for the given problems.

This paper is arranged as follows. In Section 2, we review our motivation, review the MPRP method of [33], and introduce our new PRP method. The r-step linear convergence of the new PRP method with the standard Armijo line search is proven in Section 3. In Section 4, we introduce a strategy for choosing the initial step length using the Armijo or Wolfe line search and provide the steps of the new PRP method. In Section 5, the n-step quadratic convergence of the given CG algorithm is established. In Section 6, various numerical experiments are performed to test the n-step quadratic convergence of the new PRP algorithm, and its performance is compared with that of the normal CG method. The conclusion is presented in the last section.

## Motivation and Algorithm

In this section, we will provide our motivation based on the MPRP method [33] and the BFGS formulas.

### The MPRP method of [33]

Zhang et al. [33] proposed a modified PRP method (a three-term CG method) called the MPRP method. The search direction of the MPRP method is defined by

$$\begin{array}{c} d_{k}=\{\begin{array}{cc} -g_{k}, & \text{if}\;\;\;\;k=0,\\ -g_{k}+\beta_{k}^{PRP}d_{k-1}-\theta_{k}y_{k-1}, & \text{if}\;\;\;\;k\geq 1. \end{array} \end{array}$$ (5)

where *y*_*k*−1_ = *g*_*k*_ − *g*_*k*−1_,

$$\begin{array}{c} \beta_{k}^{PRP}=\frac{g_{k}^{T}y_{k-1}}{{∥g_{k-1}∥}^{2}},\;\;\;\theta_{k}=\frac{g_{k}^{T}d_{k-1}}{g_{k}^{T}y_{k-1}}\cdot \beta_{k}^{PRP}=\frac{g_{k}^{T}d_{k-1}}{{∥g_{k-1}∥}^{2}}. \end{array}$$ (6)

This method can guarantee that *d*_*k*_ provides a descent direction of *f* at *x*_*k*_, namely, *d*_*k*_ satisfies $d_{k}^{T}g_{k}=-{\left\|g_{k}\right\|}^{2}.$ Because this method is regarded as the PRP method when the exact line search technique is used, we call it the MPRP method. This method possesses global convergence with the Armijo line search but fails using the Wolfe line search because the search direction does not belong to a trust region. The numerical results thus show that this method is effective.

### Motivation based on the BFGS formula and the MPRP method

The BFGS method is one of the most effective methods for unconstrained optimization problems. Many satisfactory results concerning this method can be found in the literature (see [35–43] etc.), wherein Wei et al. [43] presented a new BFGS update generated by Taylor’s formula

$$\begin{array}{c} B_{k}=B_{k-1}-\frac{B_{k-1}s_{k-1}s_{k-1}^{T}B_{k-1}}{s_{k-1}^{T}B_{k-1}s_{k-1}}+\frac{y_{k-1}^{m}{y_{k-1}^{m}}^{T}}{s_{k-1}^{T}y_{k-1}^{m}}, \end{array}$$

with $y_{k-1}^{m}=y_{k-1}+\frac{\rho_{k-1}}{{\left\|s_{k-1}\right\|}^{2}}s_{k-1}$ and *ρ*_*k*−1_ = 2[*f*_*k*−1_ − *f*_*k*_] + (*g*_*k*_ + *g*_*k*−1_)^*T*^ *s*_*k*−1_; *B*_*k*_ is an approximation of the Hessian matrix of the objective function *f*, with *B*_0_ = *I*. Clearly, the formula contains not only gradient value information but also function value information. Then, it might be argued that the resulting methods will substantially outperform the original method in theory. Practical computations demonstrate that this method is better than the normal BFGS method [43, 44]. Based on this method, to obtain the global convergence and the superlinear convergence of generally convex functions, Yuan and Wei [45] proposed a new quasi-Newton equation with

$$\begin{array}{c} B_{k}s_{k-1}=y_{k-1}^{*}, \end{array}$$

where $y_{k-1}^{*}=y_{k-1}+\frac{\text{max}\{\rho_{k-1},\;\;0\}}{{\left\|s_{k-1}\right\|}^{2}}s_{k-1}$ and

$$\begin{array}{c} B_{k}=B_{k-1}-\frac{B_{k-1}s_{k-1}s_{k-1}^{T}B_{k-1}}{s_{k-1}^{T}B_{k-1}s_{k-1}}+\frac{y_{k-1}^{*}{\left(y_{k-1}^{*}\right)}^{T}}{s_{k-1}^{T}y_{k-1}^{*}}, \end{array}$$ (7)

can ensure that *B*_*k*_ inherits the positive definiteness of *B*_*k*−1_ for generally convex functions; the numerical results show that this method is better than the normal CG method.

Motivated by the above discussions and the CG Eq (5), the modified PRP formula is used to replace *y*_*k*−1_ by $y_{k-1}^{*}$ in Eq (5). Specifically, the new CG formula is defined by

$$\begin{array}{c} d_{k}^{*}=\{\begin{array}{cc} -g_{k}, & \text{if}\;\;\;\;k=0,\\ -g_{k}+\beta_{k}^{{PRP}^{*}}d_{k-1}^{*}-\theta_{k}^{*}y_{k-1}^{*}, & \text{if}\;\;\;\;k\geq 1, \end{array} \end{array}$$ (8)

where

$$\begin{array}{c} y_{k-1}^{*}=y_{k-1}+\frac{\max\{\rho_{k-1},0\}}{∥s_{k-1}∥}s_{k-1},\;\;\;\;s_{k-1}=\alpha_{k-1}d_{k-1}^{*} \end{array}$$

$$\begin{array}{c} \beta_{k}^{{PRP}^{*}}=\frac{g_{k}^{T}y_{k-1}^{*}}{{∥g_{k-1}∥}^{2}},\;\;\;\;\;\theta_{k}^{*}=\frac{g_{k}^{T}d_{k-1}^{*}}{g_{k}^{T}y_{k-1}^{*}}\cdot \beta_{k}^{{PRP}^{*}}=\frac{g_{k}^{T}d_{k-1}^{*}}{{∥g_{k-1}∥}^{2}} \end{array}$$ (9)

We call this the MPRP* formula. Using (8), we can obtain

$$\begin{array}{c} d_{k}^{*T}g_{k}=-{∥g_{k}∥}^{2} \end{array}$$ (10)

and

$$\begin{array}{c} ∥g_{k}∥\;\leq \;∥d_{k}^{*}∥. \end{array}$$ (11)

## Linear convergence of the MPRP* method

The Armijo line search finds a step length *α*_*k*_ = max{*ρ* ^*i*^∣*i* = 0, 1, 2, ⋯} that satisfies

$$\begin{array}{c} f\left(x_{k}+\alpha_{k}d_{k}^{*}\right)\leq f\left(x_{k}\right)+\sigma_{1}\alpha_{k}g_{k}^{T}d_{k}^{*}, \end{array}$$ (12)

where *ρ* ∈ (0,1) and $\sigma_{1}\in (0,\frac{1}{2})$. The Wolfe line search conditions are

$$\begin{array}{c} \left\{ \begin{array}{c} f\left(x_{k}+\alpha_{k}d_{k}^{*}\right)\leq f\left(x_{k}\right)+\sigma_{1}\alpha_{k}g_{k}^{T}d_{k}^{*}\\ g{\left(x_{k}+\alpha_{k}d_{k}^{*}\right)}^{*}d_{k}^{*}\geq \sigma_{2}g_{k}^{T}d_{k}^{*}, \end{array}\right. \end{array}$$ (13)

where $0<\sigma_{1}<\frac{1}{2}$ and *σ*_1_ < *σ*_2_ < 1.

To establish the r-linear convergence of the MPRP* method, we make the following assumptions.

**Assumption (i)** *f* is a twice continuously differentiable, uniformly convex function, which means that there are positive constants *M* ≥ *m* > 0 such that

$$\begin{array}{c} m{∥d∥}^{2}\leq d^{T}\nabla^{2}f\left(x\right)d\leq M{∥d∥}^{2},\;\;\;\forall x,d\in {ℜ}^{n}, \end{array}$$

where ∇^2^ *f*(*x*) denotes the Hessian of *f* at *x*.

Under Assumption (i), it is not difficult to obtain

$$\begin{array}{c} \frac{1}{2}m{∥x-x^{*}∥}^{2}\leq f\left(x\right)-f\left(x^{*}\right)\leq \frac{1}{2}M{∥x-x^{*}∥}^{2},\;\;\;\forall x\in {ℜ}^{n} \end{array}$$ (14)

and

$$\begin{array}{c} m∥x_{k}-x^{*}∥\;\leq \;∥g_{k}∥\leq M∥x_{k}-x^{*}∥, \end{array}$$ (15)

where x* denotes the unique solution of the problem (1). Based on Assumption (i), we can easily obtain the following lemma and thus omit its proof.

**Lemma 0.1** *Let Assumption (i) hold, and let the sequence* {*x*_*k*_} *be generated by the* MPRP* *method with the Armijo or Wolfe line search. We have*

$$\begin{array}{c} -\sum_{k=0}^{\infty }g_{k}^{T}s_{k}^{*}\;<\;\infty ,\;\;\;\sum_{k=0}^{\infty }∥s_{k}^{*}∥<\infty ,\;\;\;c_{1}\alpha_{k}{∥d_{k}^{*}∥}^{2}\leq -g_{k}^{T}d_{k}^{*}, \end{array}$$

*where* $s_{k}^{*}=x_{k+1}-x_{k}$ *and* $c_{1}=\frac{1}{2}{\left(1-\sigma_{1}\right)}^{-1}m$. *Moreover, when the Wolfe line search is used, we obtain*

$$\begin{array}{c} -g_{k}^{T}d_{k}^{*}\leq {\left(1-\sigma_{2}\right)}^{-1}M\alpha_{k}{∥d_{k}^{*}∥}^{2}. \end{array}$$

**Lemma 0.2** *Let Assumption (i) hold, and let the sequence* {*x*_*k*_} *be generated by the* MPRP* *method with the Armijo line search or the Wolfe line search. Then, there is a constant* *c* > 0 *satisfying*

$$\begin{array}{c} \alpha_{k}>c,\;\;\;\forall k\geq 0. \end{array}$$ (16)

**Proof.** Let

$$\begin{array}{c} G_{k-1}=\int_{0}^{1}f\left(x_{k-1}+\tau s_{k-1}\right)d\tau , \end{array}$$

where $s_{k-1}^{*}=x_{k}-x_{k-1}=\alpha_{k-1}d_{k-1}^{*},\;\;\;y_{k-1}^{*}=y_{k-1}+\frac{\text{max}\{\rho_{k-1},0\}}{{\left\|s_{k-1}^{*}\right\|}^{2}}\cdot s_{k-1}^{*}.$ Using the definition of $\beta_{k}^{PRP^{*}},$ we obtain

$$\begin{array}{c} |\beta_{k}^{PRP^{*}}|=\frac{|g_{k}^{T}y_{k-1}^{*}|}{{∥g_{k-1}∥}^{2}}. \end{array}$$

Case 1: If *ρ*_*k*−1_ ≤ 0, we have $y_{k-1}^{*}=g_{k}-g_{k-1}$.

Using the mean-value theorem, we have

$$\begin{array}{c} y_{k-1}^{*}=g_{k}-g_{k-1}=G_{k-1}\cdot s_{k-1}^{*}=\alpha_{k-1}G_{k-1}d_{k-1}^{*}, \end{array}$$

and

$$\begin{array}{ccc} |\beta_{k}^{PRP^{*}}| & = & \frac{|g_{k}^{T}\left(g_{k}-g_{k-1}\right)|}{{∥g_{k-1}∥}^{2}}=\frac{|\alpha_{k-1}g_{k}^{T}G_{k-1}d_{k-1}^{*}|}{-g_{k-1}^{T}d_{k-1}}\\ & \leq & \frac{|\alpha_{k-1}|\cdot ∥g_{k}∥\cdot ∥G_{k-1}∥\cdot ∥d_{k-1}^{*}∥|}{c_{1}\alpha_{k-1}{∥d_{k-1}^{*}∥}^{2}}\\ & \leq & Mc_{1}^{-1}\frac{∥g_{k}∥}{∥d_{k-1}^{*}∥}. \end{array}$$

Moreover, we obtain

$$\begin{array}{c} |\theta_{k}^{*}|=\frac{|g_{k}^{T}d_{k-1}^{*}|}{{∥g_{k-1}∥}^{2}}=\frac{|g_{k}^{T}d_{k-1}^{*}|}{-g_{k-1}^{T}d_{k-1}^{*}}\leq c_{1}^{-1}\alpha_{k-1}^{-1}\frac{∥g_{k}∥}{∥d_{k-1}^{*}∥}. \end{array}$$

Thus, it follows from (8) and the Lipschitz continuity of *g* that

$$\begin{array}{ccc} ∥d_{k}^{*}∥ & = & ∥-g_{k}+\beta_{k}^{PRP^{*}}d_{k-1}^{*}-\theta_{k}^{*}y_{k-1}^{*}∥\\ & \leq & ∥g_{k}∥+|\beta_{k}^{PRP^{*}}|\cdot ∥d_{k-1}^{*}∥+|\theta_{k}^{*}|\cdot ∥y_{k-1}^{*}∥\\ & \leq & ∥g_{k}∥+\;Mc_{1}^{-1}∥g_{k}∥+\;c_{1}^{-1}\alpha_{k-1}^{-1}\cdot \frac{∥g_{k}∥}{∥d_{k-1}^{*}∥}\cdot ∥y_{k-1}^{*}∥\\ & \leq & \left(1+2Mc_{1}^{-1}\right)∥g_{k}∥. \end{array}$$

Case 2: If *ρ*_*k*−1_ > 0, we have

$$\begin{array}{ccc} y_{k-1}^{*} & = & y_{k-1}+\frac{\rho_{k-1}^{T}s_{k-1}^{*}}{{∥s_{k-1}^{*}∥}^{2}}\\ & = & \left(g_{k}-g_{k-1}\right)+\frac{\left[2\left(f\left(x_{k-1}\right)-f\left(x_{k}\right)\right)+\left(g\left(x_{k}\right)+g^{T}\left(x_{k-1}\right)\cdot s_{k-1}^{*}\right)\right]\cdot s_{k-1}^{*}}{{∥s_{k-1}^{*}∥}^{2}}\\ & = & \left(g_{k}-g_{k-1}\right)+\frac{\left(f\left(x_{k-1}\right)-f\left(x_{k}\right)\right)\cdot s_{k-1}^{*}}{{∥s_{k-1}^{*}∥}^{2}}+\left(g_{k}+g_{k-1}\right)\\ & = & 2g_{k}+\frac{\left(f\left(x_{k-1}\right)-f\left(x_{k}\right)\right)\cdot s_{k-1}^{*}}{{∥s_{k-1}^{*}∥}^{2}}. \end{array}$$

By

$$\begin{array}{c} f\left(x_{k-1}\right)-f\left(x_{k}\right)\leq \frac{1}{2}M{∥x_{k-1}-x_{k}∥}^{2}=\frac{1}{2}M{∥x_{k}-x_{k-1}∥}^{2}=\frac{1}{2}M\alpha_{k-1}^{2}{∥d_{k-1}^{*}∥}^{2} \end{array}$$

and

$$\begin{array}{c} s_{k-1}^{*}=\alpha_{k-1}\cdot d_{k-1}^{*},\;\;\;∥g_{k}∥\;\leq \;M∥x_{k}-x_{k-1}∥=M\alpha_{k-1}∥d_{k-1}^{*}∥, \end{array}$$

we obtain

$$\begin{array}{ccc} ∥y_{k-1}^{*}∥ & \leq & 2∥g_{k}+\frac{∥f\left(x_{k-1}-f\left(x_{k}\right)\right)∥\cdot ∥s_{k-1}^{*}∥}{s_{k-1}^{*}}∥\\ & \leq & 2(M\alpha_{k-1}∥d_{k-1}^{*}∥+\frac{\frac{1}{2}M\alpha_{k-1}^{2}{∥d_{k-1}^{*}∥}^{2}\alpha_{k-1}∥d_{k-1}^{*}∥}{\alpha_{k-1}^{2}{∥d_{k-1}^{*}∥}^{2}})\\ & = & 3M\alpha_{k-1}∥d_{k-1}^{*}∥. \end{array}$$

Then, we have

$$\begin{array}{c} \left|\beta_{k}^{PRP^{*}}\right|=\frac{|g_{k}^{T}y_{k-1}^{*}|}{{∥g_{k-1}∥}^{2}}\leq \frac{∥g_{k}^{T}∥\cdot ∥y_{k-1}^{*}∥}{{∥g_{k-1}∥}^{2}}\leq \frac{3M\alpha_{k-1}\cdot ∥g_{k}^{T}∥\cdot ∥d_{k-1}^{*}∥}{c_{1}\alpha_{k-1}\cdot {∥d_{k-1}^{*}∥}^{2}}\leq 3Mc_{1}^{-1}\frac{∥g_{k}∥}{∥d_{k-1}^{*}∥} \end{array}$$

and

$$\begin{array}{c} \left|\theta_{k}^{*}\right|=\frac{|g_{k}^{T}d_{k-1}^{*}|}{{∥g_{k-1}∥}^{2}}=\frac{|g_{k}^{T}d_{k-1}^{*}|}{-g_{k-1}^{T}d_{k-1}^{*}}\leq c_{1}^{-1}\alpha_{k-1}^{-1}\frac{∥g_{k}∥}{∥d_{k-1}^{*}∥}. \end{array}$$

Therefore, it follows from (8) and the Lipschitz continuity of *g* that

$$\begin{array}{ccc} ∥d_{k}^{*}∥ & = & ∥-g_{k}+\beta_{k}^{PRP^{*}}d_{k-1}^{*}-\theta_{k}^{*}y_{k-1}^{*}∥\\ & \leq & ∥g_{k}∥+\;|\beta_{k}^{PRP^{*}}|\cdot ∥d_{k-1}^{*}∥+|\theta_{k}^{*}|\cdot ∥y_{k-1}^{*}∥\\ & \leq & ∥g_{k}∥+\;\frac{3M\alpha_{k-1}∥g_{k}^{T}∥\cdot ∥d_{k-1}^{*}∥}{c_{1}\alpha_{k-1}\cdot {∥d_{k-1}^{*}∥}^{2}}\cdot ∥d_{k-1}^{*}∥+\;c_{1}^{-1}\alpha_{k-1}^{-1}\frac{∥g_{k}∥}{∥d_{k-1}^{*}∥}\cdot ∥y_{k-1}^{*}∥\\ & \leq & ∥g_{k}∥+\;3c_{1}^{-1}M∥g_{k}∥+\;c_{1}^{-1}\alpha_{k-1}^{-1}\frac{∥g_{k}∥}{∥d_{k-1}^{*}∥}\cdot 3M\alpha_{k-1}∥d_{k-1}^{*}∥\\ & \leq & \left(1+6c_{1}^{-1}M\right)∥g_{k}∥. \end{array}$$ (17)

If the Armijo line search is used, and *α*_*k*_ ≠ 1, then $\alpha_{k}^{\prime }=\alpha_{k}\rho^{-1}$ such that $f(x_{k}+\alpha_{k}^{\prime }d_{k}^{*})-f(x_{k})>\sigma_{1}\alpha_{k}^{\prime }g_{k}^{T}d_{k}^{*}.$ By the mean-value theorem and the above relation, we can deduce that there exists a scalar *μ*_*k*_ ∈ (0,1) satisfying

$$\begin{array}{ccc} \sigma_{1}\alpha_{k}^{{}^{\prime }}g_{k}^{T}d_{k}^{*} & < & f\left(x_{k}+\alpha_{k}^{{}^{\prime }}d_{k}^{*}\right)-f\left(x_{k}\right)=\alpha_{k}^{{}^{\prime }}g{\left(x_{k}+\mu_{k}\alpha_{k}^{{}^{\prime }}d_{k}^{*}\right)}^{T}d_{k}^{*}\\ & = & \alpha_{k}^{{}^{\prime }}{\left(g\left(x_{k}+\mu_{k}\alpha_{k}^{{}^{\prime }}d_{k}^{*}\right)-g\left(x_{k}\right)\right)}^{T}d_{k}^{*}+\alpha_{k}^{{}^{\prime }}g_{k}^{T}d_{k}^{*}\\ & = & \mu_{k}{\left(\alpha_{k}^{{}^{\prime }}\right)}^{2}d_{k}^{*T}\int_{0}^{1}\nabla^{2}f\left(x_{k}+\tau \mu_{k}\alpha_{k}^{{}^{\prime }}d_{k}^{*}\right)d\tau +\alpha_{k}^{{}^{\prime }}g_{k}^{T}d_{k}^{*}\\ & \leq & {\left(\alpha_{k}^{{}^{\prime }}\right)}^{2}M\cdot {∥d_{k}^{*}∥}^{2}+\alpha_{k}^{{}^{\prime }}g_{k}^{T}d_{k}^{*}. \end{array}$$

Thus, the relation

$$\begin{array}{c} \alpha_{k}=\rho \alpha_{k}^{{}^{\prime }}\geq -\frac{(1-\sigma_{1})\rho }{M}\cdot \frac{g_{k}^{T}d_{k}^{*}}{{∥d_{k}^{*}∥}^{2}}=\frac{(1-\sigma_{1})\rho }{M}\cdot \frac{{∥g_{k}∥}^{2}}{{∥d_{k}∥}^{2}} \end{array}$$

holds. By the relation $1+6Mc_{1}^{-1}>1+2Mc_{1}^{-1}>0$, we can find that

$$\begin{array}{c} \frac{1}{1+2Mc_{1}^{-1}}>\frac{1}{1+6Mc_{1}^{-1}}. \end{array}$$

Then, we obtain

$$\begin{array}{c} \alpha_{k}=\rho \alpha_{k}^{{}^{\prime }}\geq -\frac{(1-\sigma_{1})\rho }{M}\cdot \frac{g_{k}^{T}d_{k}^{*}}{{∥d_{k}^{*}∥}^{2}}=\frac{(1-\sigma_{1})\rho }{M}\cdot \frac{{∥g_{k}∥}^{2}}{{∥d_{k}^{*}∥}^{2}}\geq M^{-1}\left(1-\sigma_{1}\right)\rho {\left(1+2Mc_{1}^{-1}\right)}^{-2}\equiv \bar{c}. \end{array}$$

Setting $c=\text{min}\{1,\bar{c}\}$, we have Eq (16). If the Wolfe line search is used, using Eq (13), we have

$$\begin{array}{c} M\alpha_{k}{∥d_{k}^{*}∥}^{2}\geq {\left(g\left(x_{k}+\alpha_{k}d_{k}^{*}\right)-g_{k}\right)}^{T}d_{k}^{*}\geq -\left(1-\sigma_{2}\right)g_{k}^{T}d_{k}^{*}. \end{array}$$

By analyzing the Armijo line search technique in a similar manner, we can find a lower positive bound of the step size *α*_*k*_. The proof is complete.

Similar to [34], we can establish the following theorem. Here, we state the theorem below but omit its proof.

**Theorem 0.1** *Let Assumption (i) hold, and let the sequence* {*x*_*k*_} *be generated by the* MPRP* *method with the Armijo line search technique or Wolfe line search technique. Then, there are constants* *a* > 0 *and* *r* ∈ (0,1) *that satisfy*

$$\begin{array}{c} ∥x_{k}-x^{*}∥\leq ar^{k}. \end{array}$$ (18)

## The restart MPRP* method and its convergence

As with [34], we define an initial step length $\gamma_{k}^{*}$ as follows:

$$\begin{array}{c} \gamma_{k}^{*}\equiv \frac{\epsilon_{k}{∥g_{k}∥}^{2}}{d_{k}^{*T}\left(g\left(x_{k}+\epsilon_{k}d_{k}^{*}\right)-g\left(x_{k}\right)\right)}, \end{array}$$ (19)

using the integer sequence {*ϵ*_*k*_} → 0 with *k* → ∞. Moreover, we can obtain

$$\begin{array}{c} |\bar{\alpha_{k}}-\gamma_{k}^{*}|\to 0. \end{array}$$

**Theorem 0.2** *Let sequence* {*x*_*k*_} *be generated by the* MPRP* *method, and let Assumption (i) hold. Then, for sufficiently large* *k*, $\gamma_{k}^{*}$ *satisfies the Armijo line search and the Wolfe-Powell line search conditions*.

Theorem 0.2 shows that, for sufficiently large *k*, $\gamma_{k}^{*}$ can be defined by Eq (19) such that the Armijo line search and Wolfe-Powell line search conditions are satisfied. In the following, ∣*γ*_*k*_∣ is used as the initial step length of the restart MPRP* method.

**Algorithm 4.1** (RMPRP*)

Step 0: Given $x_{0}\in {ℜ}^{n},\;\;\sigma_{1}\in (0,\frac{1}{2})$, *ρ* ∈ [0,1), *ϵ* ∈ [0,1), and *γ* > 0 is an integer, let *k*: = 0.

Step 1: If ‖*g*_*k*_‖ ≤ *ϵ*, stop.

Step 2: If the inequality $f(x_{k}+|\gamma_{k}|d_{k}^{*})\leq f(x_{k})+\sigma_{1}|\gamma_{k}|g_{k}^{T}d_{k}^{*}$ holds, we set *α*_*k*_ = ∣*γ*_*k*_∣; otherwise, we determine *α*_*k*_ = max{∣*γ*_*k*_∣*ρ* ^*j*^∣*j* = 0, 1, 2, ⋯} satisfying

$$\begin{array}{c} f\left(x_{k}+\alpha_{k}d_{k}^{*}\right)\leq f\left(x_{k}\right)+\sigma_{1}\alpha_{k}g_{k}^{T}d_{k}^{*}. \end{array}$$ (20)

Step 3: Let $x_{k+1}=x_{k}+\alpha_{k}d_{k}^{*}$, and *k*: = *k*+1.

Step 4: If ‖*g*_*k*_‖ ≤ *ϵ*, stop.

Step 5: If *k* = *γ*, we let *x*_0_: = *x*_*k*_. Go to step 1.

Step 6: Compute $d_{k}^{*}$ using (8). Go to step 2.

We now establish the global convergence of Algorithm 4.1.

**Theorem 0.3** *Let the conditions of Theorem 0.2 hold. Then, the following relation*

$$\begin{array}{c} \lim_{k\to \infty }∥g_{k}∥=0 \end{array}$$ (21)

*holds*.

**Proof.** We will prove this theorem by contradiction. Suppose that Eq (21) does not hold; then, for all *k*, there exists a constant *ɛ*_1_ > 0 satisfying

$$\begin{array}{c} ∥g_{k}∥\geq \;\epsilon_{1}. \end{array}$$ (22)

Using Eqs (10) and (20), if *f* is bounded from below, we can obtain

$$\begin{array}{c} \sum_{k=0}^{\infty }\alpha_{k}^{2}{∥d_{k}^{*}∥}^{2}<\infty . \end{array}$$ (23)

In particular, we have

$$\begin{array}{c} \lim_{k\to \infty }\alpha_{k}∥d_{k}^{*}∥=0. \end{array}$$ (24)

If lim_*k* → ∞_ *α*_*k*_ > 0, by Eqs (10) and (24), we obtain lim_*k* → ∞_‖*g*_*k*_‖ = 0. This contradicts Eqs (22) and (21) holds. Otherwise, if lim_*k* → ∞_ *α*_*k*_ = 0., then there is an infinite index set *K*_0_ satisfying

$$\begin{array}{c} \lim_{k\in K_{0},\;k\to \infty }\alpha_{k}=0. \end{array}$$ (25)

According to Step 2 of Algorithm 4.1, when *k* is sufficiently large, *α*_*k*_ *ρ* ^−1^ does not satisfy Eq (20), which implies that

$$\begin{array}{c} f\left(x_{k}+\alpha_{k}\rho^{-1}d_{k}^{*}\right)-f\left(x_{k}\right)>-\delta \rho^{-2}\alpha_{k}^{2}{∥d_{k}^{*}∥}^{2}. \end{array}$$ (26)

By Eq (22), similar to the proof of Lemma 2.1 in [33], we can deduce that there is a constant *ϱ* > 0 such that

$$\begin{array}{c} ∥d_{k}^{*}∥\leq \varrho ,\;\;\forall \;\;k. \end{array}$$ (27)

Using Eqs (27) and (10), and the mean-value theorem, we have

$$\begin{array}{ccc} f\left(x_{k}+\alpha_{k}\rho^{-1}d_{k}^{*}\right)-f\left(x_{k}\right) & = & \rho^{-1}\alpha_{k}g{\left(x_{k}+\xi_{0}\rho^{-1}\alpha_{k}d_{k}^{*}\right)}^{T}d_{k}^{*}\\ & = & \rho^{-1}\alpha_{k}g_{k}^{T}d_{k}^{*}+\rho^{-1}\alpha_{k}{\left(g\left(x_{k}+\xi_{0}\rho^{-1}\alpha_{k}d_{k}^{*}\right)-g_{k}\right)}^{T}d_{k}^{*}\\ & \leq & \rho^{-1}\alpha_{k}g_{k}^{T}d_{k}^{*}+M\rho^{-2}\alpha_{k}^{2}{∥d_{k}^{*}∥}^{2}, \end{array}$$

where *ξ*_0_ ∈ (0,1) and the last inequality follows Eq (15). Combining this result with Eq (26), for all sufficiently large *k* ∈ *K*_0_, we obtain

$$\begin{array}{c} {∥g_{k}∥}^{2}\leq \rho^{-1}\left(M+\delta \right)\alpha_{k}{∥d_{k}^{*}∥}^{2}. \end{array}$$

By Eq (27) and lim_*k* → ∞_ *α*_*k*_ = 0, the above inequality then implies that lim_*k* ∈ *K*_0_, *k* → ∞_‖*g*_*k*_‖ = 0. This is also a contradiction. The proof is complete.

**Lemma 0.3** *Let Assumption (i) hold, and let the sequence* {*x*_*k*_} *be generated by the* RMPRP* *method. Then, there are four positive numbers* *c*_*i*_, *i* = 1, 2, 3, 4 *that satisfy*

$$\begin{array}{c} ∥g_{k+1}∥\leq c_{1}∥d_{k}^{*}∥,\;\;\;|\beta_{k+1}^{PRP^{*}}|\leq c_{2},\;\;\;|\theta_{k+1}^{*}|\leq c_{3},\;\;\;∥d_{k+1}^{*}∥\leq c_{4}∥d_{k}^{*}∥. \end{array}$$ (28)

**Proof.** Considering the first inequality of Eq (28), we have

$$\begin{array}{ccc} ∥g_{k+1}∥ & = & ∥g_{k}+\left(g_{k+1}-g_{k}\right)∥\;\leq \;∥g_{k}∥+\;\left|\gamma_{k}\right|\;\cdot ∥\hat{A_{k}}d_{k}^{*}∥\\ & \leq & ∥d_{k}^{*}∥+\;\frac{M}{m}\cdot ∥d_{k}^{*}∥=\left(1+\frac{M}{m}\right)∥d_{k}^{*}∥\equiv c_{1}∥d_{k}^{*}∥, \end{array}$$ (29)

where $\hat{A_{k}}=\int_{0}^{1}\nabla^{2}f(x_{k}+\tau |\gamma_{k}|d_{k}^{*})d\tau$. Using $\beta_{k+1}^{PRP^{*}},$ we will discuss the three other inequalities of Eq (28). For $\beta_{k+1}^{PRP^{*}}$, we have

$$\begin{array}{c} \left|\beta_{k+1}^{PRP^{*}}\right|=\frac{|g_{k+1}^{T}y_{k}^{*}|}{{∥g_{k}∥}^{2}}\leq \frac{∥g_{k+1}^{T}∥\cdot ∥y_{k}^{*}∥}{{∥g_{k}∥}^{2}}. \end{array}$$

Case 1: If *ρ*_*k*_ ≤ 0, then $y_{k}^{*}=y_{k}=g_{k+1}-g_{k}$. Therefore, we have

$$\begin{array}{ccc} |\beta_{k+1}^{PRP^{*}}| & \leq & \frac{∥g_{k+1}∥}{{∥g_{k}∥}^{2}}∥g_{k+1}-g_{k}∥\leq \frac{c_{1}\left|\gamma_{k}\right|{∥d_{k}^{*}∥}^{2}M}{{∥g_{k}∥}^{2}}\\ & = & \frac{c_{1}{∥d_{k}^{*}∥}^{2}M}{d_{k}^{*T}A_{k}d_{k}^{*}}\leq \frac{c_{1}{∥d_{k}^{*}∥}^{2}M}{d_{k}^{*T}A_{k}d_{k}^{*}}\leq c_{1}m^{-1}M\equiv \bar{c_{2}} \end{array}$$

where $A_{k}=\int_{0}^{1}\nabla^{2}f(x_{k}+\tau \epsilon_{k}d_{k}^{*})d\tau$ and the second inequality follows from the mean-value theorem and Eq (29).

Case 2: If *ρ*_*k*_ > 0, then we have

$$\begin{array}{c} \left\{ \begin{array}{c} y_{k}^{*}=y_{k}+\frac{\rho_{k}^{T}s_{k}^{*}}{{∥s_{k}^{*}∥}^{2}}\\ \rho_{k}=2\left[f\left(x_{k}\right)-f\left(x_{k+1}\right)\right]+{\left(g\left(x_{k+1}\right)+g\left(x_{k}\right)\right)}^{T}s_{k}^{*}. \end{array}\right. \end{array}$$

Thus, we have

$$\begin{array}{ccc} |\beta_{k+1}^{PRP^{*}}| & \leq & \frac{∥g_{k+1}^{T}∥\cdot ∥y_{k}^{*}∥}{{∥g_{k}∥}^{2}}\\ & \leq & \frac{∥g_{k+1}∥}{{∥g_{k}∥}^{2}}[∥g_{k+1}-g_{k}∥+\frac{2∥f\left(x_{k+1}\right)-f\left(x_{k}\right)∥\cdot ∥s_{k}^{*}∥+∥g_{k+1}+g_{k}∥\cdot {∥s_{k}^{*}∥}^{2}}{{∥s_{k}^{*}∥}^{2}}]\\ & \leq & \frac{∥g_{k+1}^{T}∥\cdot ∥g_{k+1}-g_{k}∥}{{∥g_{k}∥}^{2}}+\frac{∥g_{k+1}∥}{{∥g_{k}∥}^{2}}\cdot \frac{2∥f\left(x_{k+1}\right)-f\left(x_{k}\right)∥+∥g_{k+1}+g_{k}∥∥s_{k}^{*}∥}{∥s_{k}^{*}∥}\\ & \leq & \bar{c_{2}}+\frac{∥g_{k+1}∥}{{∥g_{k}∥}^{2}}\left(\frac{2∥f\left(x_{k+1}\right)-f\left(x_{k}\right)∥}{∥s_{k}^{*}∥}+∥g_{k+1}+g_{k}∥\right)\\ & \leq & \frac{c_{1}∥d_{k}^{*}∥}{{\left(1+2Mc_{1}^{-1}\right)}^{-2}{∥d_{k}^{*}∥}^{2}}\left[\frac{M\cdot {∥s_{k}^{*}∥}^{2}}{∥s_{k}^{*}∥}+\left(1+c_{1}\right)∥d_{k}^{*}∥\right]+\bar{c_{2}}\\ & \leq & \bar{c_{2}}+\left[c_{1}{\left(1+2Mc_{1}^{-1}\right)}^{2}\left(M+c_{1}+1\right)\right]\equiv \bar{\bar{c_{2}}}. \end{array}$$

Let $c_{2}=\text{max}\{\bar{c_{2}},\bar{\bar{c_{2}}}\}$. We obtain

$$\begin{array}{c} |\beta_{k+1}^{PRP^{*}}|\leq c_{2} \end{array}$$

and

$$\begin{array}{c} \left|\theta_{k+1}^{*}\right|=\left|\frac{g_{k+1}^{T}d_{k}^{*}}{{∥g_{k}∥}^{2}}\right|\leq ∥g_{k+1}∥\cdot \frac{∥d_{k}^{*}∥}{{∥g_{k}∥}^{2}}\leq c_{1}\frac{{∥d_{k}^{*}∥}^{2}}{{∥g_{k}∥}^{2}}\leq c_{1}{\left(1+2Mc_{1}^{-1}\right)}^{2}\equiv c_{3}. \end{array}$$

Using the definition of $d_{k+1}^{*}$, we obtain

$$\begin{array}{ccc} ∥d_{k+1}^{*}∥ & = & ∥-g_{k+1}+\beta_{k+1}^{PRP^{*}}d_{k}^{*}-\theta_{k+1}^{*}y_{k}^{*}∥\\ & \leq & ∥g_{k+\;1}∥+\;|\beta_{k+1}^{PRP^{*}}|\cdot ∥d_{k}^{*}∥+|\theta_{k+1}^{*}|\cdot ∥y_{k+1}^{*}∥. \end{array}$$

Case 1: If *ρ*_*k*_ ≤ 0, we have

$$\begin{array}{ccc} ∥d_{k+1}^{*}∥ & \leq & ∥g_{k+1}∥+\;|\beta_{k+1}^{PRP^{*}}|\cdot ∥d_{k}^{*}∥+\;|\theta_{k+1}^{*}|(∥g_{k+1}+∥g_{k}∥∥)\\ & = & \left(1+\right|\theta_{k+1}^{*}|)∥g_{k+1}∥+\;|\beta_{k+1}^{PRP^{*}}|\cdot ∥d_{k}^{*}∥+|\theta_{k+1}^{*}|\cdot ∥g_{k}∥\\ & \leq & \left(1+c_{3}\right)c_{1}∥d_{k}^{*}∥+\;c_{2}∥d_{k}^{*}∥+c_{3}∥d_{k}^{*}∥\\ & = & \left[\left(1+c_{3}\right)c_{1}+c_{2}+c_{3}\right]\cdot ∥d_{k}^{*}∥\equiv \bar{c_{4}}∥d_{k}^{*}∥. \end{array}$$

Case 2: If *ρ*_*k*_ > 0, we have

$$\begin{array}{c} ∥y_{k}^{*}∥\leq 3M\alpha_{k}∥d_{k}^{*}∥\;\leq \;3M∥d_{k}^{*}∥,\;\;\;\left(0<\alpha_{k}\leq 1\right), \end{array}$$

and

$$\begin{array}{ccc} ∥d_{k+1}^{*}∥ & \leq & ∥g_{k+1}∥+|\beta_{k+1}^{PRP^{*}}|\cdot ∥d_{k}^{*}∥+|\theta_{k+1}^{*}|\cdot ∥y_{k}^{*}∥\\ & \leq & c_{1}∥d_{k}^{*}∥+c_{2}∥d_{k}^{*}∥+3c_{3}M∥d_{k}^{*}∥\\ & = & \left(c_{1}+c_{2}+3Mc_{3}\right)∥d_{k}^{*}∥\equiv \bar{\bar{c_{4}}}∥d_{k}^{*}∥. \end{array}$$

Letting $c_{4}=\text{max}\{\bar{c_{4}},\bar{\bar{c_{4}}}\}$, we obtain $\left\|d_{k+1}^{*}\|\leq c_{4}\|d_{k}^{*}\right\|$. The proof is complete.

**Assumption (ii)** In some neighborhood *N* of x*, ∇^2^ *f* is Lipschitz continuous.

Similar to [34], we can also establish the n-order quadratic convergence of the RMPRP* method. Here, we state the theorem but omit the proof.

**Theorem 0.4** *Let Assumptions (i) and (ii) hold. Then, there exists a constant* *c*′ > 0 *such that*

$$\begin{array}{c} \lim_{k\to \infty }sup\frac{∥x_{kr+n}-x^{*}∥}{{∥x_{kr}-x^{*}∥}^{2}}\leq c^{{}^{\prime }}<\infty . \end{array}$$ (30)

*Specifically, the* RMPRP* *method is quadratically convergent*.

## Numerical Results

This section reports on various numerical experiments with Algorithm 4.1 and the normal algorithm to demonstrate the effectiveness of the given algorithm. We utilize the normal algorithm (called **Algorithm N**), whereby the algorithm does not use the restart technique, but the other steps are the same as those in Algorithm 4.1. We will test the following benchmark problems using these two algorithms. These problems are listed in Table 1. These benchmark problems and discussions concerning the choice of tested problems for an algorithm can be found at

$$\begin{array}{c} http://www.cs.cmu.edu/afs/cs/project/jair/pub/volume24/ortizboyer05a-html/node6.html. \end{array}$$

**Table 1:** Definition of the benchmark problems and their features.

No.	Functions	Definition	Multimodal?	Separable?	Regular?
1	Sphere	$f_{Sph}(x)=\sum_{i=1}^{p}x_{i}^{2}$ *x*_*i*_ ∈ [−5.12, 5.12], x* = (0, 0, ⋯, 0), *f*_*Sph*_(x*) = 0.	no	yes	n/a
2	Schwefel’s	$f_{SchDS}(x)=\sum_{i=1}^{p}{\left(\sum_{j=1}^{i}x_{j}\right)}^{2}$ *x*_*i*_ ∈ [−65.536, 65.536], x* = (0, 0, ⋯, 0), *f*_*SchDS*_(x*) = 0.	no	no	n/a
3	Rastrigin	$f_{Ras}(x)=10p+\sum_{i=1}^{p}(x_{i}^{2}-10\text{cos}(2\pi x_{i}))$ *x*_*i*_ ∈ [−5.12, 5.12], x* = (0, 0, ⋯, 0), *f*_*Ras*_(x*) = 0.	yes	yes	n/a
4	Schwefel	$f_{Sch}(x)=418.9829p+\sum_{i=1}^{p}x_{i}\text{sin}\sqrt{|x_{i}|}$ *x*_*i*_ ∈ [−512.03, 511.97], x* = (−420.9678, −420.9678, ⋯, −420.9678), *f*_*Sch*_(x*) = 0.	yes	yes	n/a
5	Griewank	$f_{Gri}(x)=1+\sum_{i=1}^{p}\frac{x_{i}^{2}}{4000}-\prod_{i=1}^{p}\text{cos}\frac{x_{i}}{i}$ *x*_*i*_ ∈ [−600, 600], x* = (0, 0, ⋯, 0), *f*_*Gri*_(x*) = 0.	yes	no	yes
6	Rosenbrock	$f_{Ros}(x)=\sum_{i=1}^{p-1}[100{\left(x_{i+1}-x_{i}^{2}\right)}^{2}+{\left(x_{i}-1\right)}^{2}]$ *x*_*i*_ ∈ [−2.048, 2.048], x* = (1, ⋯, 1), *f*_*Ros*_(x*) = 0.	no	no	n/a
7	Ackley	$f_{Ack}(x)=20+e-20e^{-0.2\sqrt{\frac{1}{p}\sum_{i=1}^{p}x_{i}^{2}}}-e^{\frac{1}{p}\sum_{i=1}^{p}\text{cos}(2\pi x_{i})}$ *x*_*i*_ ∈ [−30, 30], x* = (0, ⋯, 0), *f*_*Ack*_(x*) = 0.	yes	no	yes
8	Langerman	$f_{Lan}(x)=-\sum_{i=1}^{m}c_{i}e^{-\frac{1}{\pi }\sum_{j=1}^{p}{\left(x_{j}-a_{ij}\right)}^{2}}\text{cos}(\pi \sum_{j=1}^{p}{\left(x_{j}-a_{ij}\right)}^{2})$ *x*_*i*_ ∈ [0, 10], *m* = *p*, x* = *random*, *f*_*Lan*_(x*) = *random*.	yes	no	no

**Table 2:** Test results using Algorithm 4.1.

	Dim	NI/NFG/$f(\bar{x})$	NI/NFG/$f(\bar{x})$	NI/NFG/$f(\bar{x})$	NI/NFG/$f(\bar{x})$
No.	*x*_0_	(−5, ⋯)	(−3, ⋯)	(2, ⋯)	(4, ⋯)
1	30	2/6/8.355554e-023	2/6/3.274972e-024	2/6/1.455543e-024	2/6/5.822172e-024
	10000	2/6/2.788150e-020	2/6/1.091657e-021	2/6/4.851810e-022	2/6/1.940724e-021
	100000	2/6/5.970100e-019	2/6/8.813541e-020	2/6/4.871394e-021	2/6/1.948557e-020
	103000	2/6/5.962874e-019	2/6/9.164034e-020	2/6/4.997364e-021	2/6/1.998946e-020
	*x*_0_	(−0.002, ⋯)	(−0.001, ⋯)	(0.001, ⋯)	(0.0002, ⋯)
2	30	4/12/7.338081e-006	4/12/1.834520e-006	4/12/1.834520e-006	3/9/4.070200e-007
	60	5/15/1.607584e-005	4/12/1.503191e-005	4/12/1.503191e-005	3/9/3.227252e-006
	100	6/18/2.574911e-005	5/15/1.889258e-005	5/15/1.889258e-005	4/12/2.785399e-006
	200	8/24/3.830680e-005	7/21/2.110989e-005	7/21/2.110989e-005	5/15/6.065297e-006
	*x*_0_	(−0.02, 0, ⋯)	(0.01, 0, ⋯)	(0.001, 0, ⋯)	(0.006, 0, ⋯)
3	30	3/9/0.000000e+000	3/9/0.000000e+000	2/6/4.547474e-013	3/9/0.000000e+000
	60	3/9/0.000000e+000	3/9/0.000000e+000	2/6/1.136868e-012	3/9/0.000000e+000
	100	3/9/0.000000e+000	3/9/0.000000e+000	2/6/1.136868e-012	3/9/0.000000e+000
	200	3/9/0.000000e+000	3/9/0.000000e+000	2/6/1.136868e-012	3/9/0.000000e+000
	*x*_0_	(−500, ⋯)	(−10, ⋯)	(−300, ⋯)	(50, ⋯)
4	30	9/28/4.588939e+002	5/15/1.233277e+004	10/30/8.751388e+003	2/16/1.469607e+004
	1000	9/28/1.529646e+004	5/15/4.110923e+005	10/30/2.917129e+005	2/16/4.898690e+005
	10000	9/28/1.529646e+005	5/15/4.110923e+006	10/30/2.917129e+006	2/16/4.898690e+006
	100000	9/28/1.529646e+006	5/15/4.110923e+007	10/30/2.917129e+007	2/16/4.898690e+007
	*x*_0_	(−2, ⋯)	(−1, ⋯)	(1, ⋯)	(2, ⋯)
5	30	2/6/8.548717e-015	2/6/5.644248e-009	2/6/5.644248e-009	2/6/8.548717e-015
	1000	2/6/0.000000e+000	2/6/0.000000e+000	2/6/0.000000e+000	2/6/0.000000e+000
	10000	2/6/0.000000e+000	2/6/0.000000e+000	2/6/0.000000e+000	2/6/0.000000e+000
	100000	2/6/0.000000e+000	2/6/0.000000e+000	2/6/0.000000e+000	2/6/0.000000e+000
	*x*_0_	(1.01, ⋯)	(0.9999, ⋯)	(1.003, ⋯)	(1.005, ⋯)
6	30	12/36/6.832422e-005	2/6/1.919312e-006	6/18/5.497859e-005	9/27/6.152662e-005
	1000	12/36/6.562848e-005	3/9/3.607120e-007	6/18/5.343543e-005	9/27/5.799691e-005
	10000	12/36/6.382394e-005	3/9/3.615221e-007	7/21/4.048983e-005	9/27/6.078016e-005
	100000	12/36/5.969884e-005	3/9/3.632823e-007	7/21/4.484866e-005	9/27/5.885821e-005
	*x*_0_	(−0.1, ⋯)	(0.1, ⋯)	(0.01, ⋯)	(0.03, ⋯)
7	30	11/50/1.684545e+000	11/50/1.684545e+000	11/58/1.684467e+000	6/25/1.684388e+000
	1000	14/67/1.717428e+000	14/67/1.717428e+000	9/46/1.717374e+000	11/56/1.717448e+000
	10000	15/75/1.718189e+000	15/75/1.718189e+000	9/46/1.718252e+000	11/57/1.718327e+000
	100000	15/75/1.718273e+000	15/75/1.718273e+000	9/46/1.718340e+000	11/57/1.718410e+000
	*x*_0_	(−5, ⋯)	(−1, ⋯)	(2, ⋯)	(4, ⋯)
8	30	1/3/-1.889333e-104	2/16/-8.226096e-004	1/3/-2.009161e-015	1/3/-1.228836e-064
	300	1/3/0.000000e+000	1/3/-3.893431e-040	1/3/-1.008253e-163	1/3/0.000000e+000
	500	1/3/0.000000e+000	1/3/-1.459270e-067	1/3/-4.297704e-274	1/3/0.000000e+000
	1000	1/3/0.000000e+000	1/3/-2.213361e-136	1/3/0.000000e+000	1/3/0.000000e+000

We will use these benchmark problems to perform the experiments with these two algorithms. The code was written in MATLAB 7.6.0 and run on a PC with a Core 2 Duo CPU, E7500 @2.93 GHz, 2.00 GB memory and Windows XP operation system. The parameters of the algorithms are chosen to be *ρ* = 0.1, *γ* = 10, *σ*_1_ = 0.0001, and *γ*_*k*_ = *e*_1_ = *ϵ* = 10^−4^. The dimension can be found in Tables 2–3. Because the line search cannot always ensure that the descent condition $d_{k}^{T}g_{k}<0$ holds, uphill searches will occur in the experiments. To avoid this situation, the step size *α*_*k*_ will be accepted if the searching number is larger than ten in every line search technique. The *Himmeblau* stop rule will be used:

If ∣*f*(*x*_*k*_)∣ > *e*_1_, set $stop1=\frac{|f(x_{k})-f(x_{k+1})|}{|f(x_{k})|};$ otherwise, set *stop*1 = ∣*f*(*x*_*k*_)−*f*(*x*_*k*+1_)∣.

For each problem, the program will stop if ‖*g*(*x*)‖ < *e*_3_ or *stop*1 < *e*_2_ is satisfied, where *e*_1_ = *e*_2_ = *e*_3_ = 10^−4^.

The program also stops if more than five thousand iterations are performed because the corresponding method for the problem is regarded as having failed. The meanings of the columns in Tables 2–3 are as follows:

*x*_0_: the initial point NFG: the total number of NF and NG, i.e., *NFG* = *NF*+*NG*.

NI: the total number of iterations Dim: the dimension of the problem;

$f(\bar{x})$ denotes the function value at the point $\bar{x}$ when the program stops.

**Table 3:** Test results using Algorithm N.

	Dim	NI/NFG/$f(\bar{x})$	NI/NFG/$f(\bar{x})$	NI/NFG/$f(\bar{x})$	NI/NFG/$f(\bar{x})$
No.	*x*_0_	(−5, ⋯)	(−3, ⋯)	(2, ⋯)	(4, ⋯)
1	30	2/6/8.355554e-023	2/6/3.274972e-024	2/6/1.455543e-024	2/6/5.822172e-024
	10000	2/6/2.788150e-020	2/6/1.091657e-021	2/6/4.851810e-022	2/6/1.940724e-021
	100000	2/6/5.970100e-019	2/6/8.813541e-020	2/6/4.871394e-021	2/6/1.948557e-020
	103000	2/6/5.962874e-019	2/6/9.164034e-020	2/6/4.997364e-021	2/6/1.998946e-020
	*x*_0_	(−0.002, ⋯)	(−0.001, ⋯)	(0.001, ⋯)	(0.0002, ⋯)
2	30	4/12/7.338081e-006	4/12/1.834520e-006	4/12/1.834520e-006	3/9/4.070200e-007
	60	5/15/1.607584e-005	4/12/1.503191e-005	4/12/1.503191e-005	3/9/3.227252e-006
	100	6/18/2.574911e-005	5/15/1.889258e-005	5/15/1.889258e-005	4/12/2.785399e-006
	200	8/24/3.830680e-005	7/21/2.110989e-005	7/21/2.110989e-005	5/15/6.065297e-006
	*x*_0_	(−0.02, 0, ⋯)	(0.01, 0, ⋯)	(0.001, 0, ⋯)	(0.006, 0, ⋯)
3	30	3/9/0.000000e+000	3/9/0.000000e+000	2/6/4.547474e-013	3/9/0.000000e+000
	60	3/9/0.000000e+000	3/9/0.000000e+000	2/6/1.136868e-012	3/9/0.000000e+000
	100	3/9/0.000000e+000	3/9/0.000000e+000	2/6/1.136868e-012	3/9/0.000000e+000
	200	3/9/0.000000e+000	3/9/0.000000e+000	2/6/1.136868e-012	3/9/0.000000e+000
	*x*_0_	(−500, ⋯)	(−10, ⋯)	(−300, ⋯)	(50, ⋯)
4	30	9/28/4.588939e+002	5/15/1.233277e+004	10/30/8.751388e+003	2/16/1.469607e+004
	1000	9/28/1.529646e+004	5/15/4.110923e+005	10/30/2.917129e+005	2/16/4.898690e+005
	10000	9/28/1.529646e+005	5/15/4.110923e+006	10/30/2.917129e+006	2/16/4.898690e+006
	100000	9/28/1.529646e+006	5/15/4.110923e+007	10/30/2.917129e+007	2/16/4.898690e+007
	*x*_0_	(−2, ⋯)	(−1, ⋯)	(1, ⋯)	(2, ⋯)
5	30	2/6/8.548717e-015	2/6/5.644248e-009	2/6/5.644248e-009	2/6/8.548717e-015
	1000	2/6/0.000000e+000	2/6/0.000000e+000	2/6/0.000000e+000	2/6/0.000000e+000
	10000	2/6/0.000000e+000	2/6/0.000000e+000	2/6/0.000000e+000	2/6/0.000000e+000
	100000	2/6/0.000000e+000	2/6/0.000000e+000	2/6/0.000000e+000	2/6/0.000000e+000
	*x*_0_	(1.01, ⋯)	(0.9999, ⋯)	(1.003, ⋯)	(1.005, ⋯)
6	30	16/48/8.156782e-005	2/6/1.919312e-006	6/18/5.497859e-005	9/27/6.152662e-005
	1000	15/45/8.236410e-005	3/9/3.607120e-007	6/18/5.343543e-005	9/27/5.799691e-005
	10000	14/42/8.568194e-005	3/9/3.615221e-007	7/21/4.048983e-005	9/27/6.078016e-005
	100000	13/39/8.458113e-005	3/9/3.632823e-007	7/21/4.484866e-005	9/27/5.885821e-005
	*x*_0_	(−0.1, ⋯)	(0.1, ⋯)	(0.01, ⋯)	(0.03, ⋯)
7	30	11/50/1.684545e+000	11/50/1.684545e+000	11/58/1.684467e+000	6/25/1.684388e+000
	1000	14/67/1.717428e+000	14/67/1.717428e+000	9/46/1.717374e+000	11/56/1.717448e+000
	10000	15/75/1.718189e+000	15/75/1.718189e+000	9/46/1.718252e+000	11/57/1.718327e+000
	100000	15/75/1.718273e+000	15/75/1.718273e+000	9/46/1.718340e+000	11/57/1.718410e+000
	*x*_0_	(−5, ⋯)	(−1, ⋯)	(2, ⋯)	(4, ⋯)
8	30	1/3/-1.889333e-104	2/16/-8.226096e-004	1/3/-2.009161e-015	1/3/-1.228836e-064
	300	1/3/0.000000e+000	1/3/-3.893431e-040	1/3/-1.008253e-163	1/3/0.000000e+000
	500	1/3/0.000000e+000	1/3/-1.459270e-067	1/3/-4.297704e-274	1/3/0.000000e+000
	1000	1/3/0.000000e+000	1/3/-2.213361e-136	1/3/0.000000e+000	1/3/0.000000e+000

The results in Tables 2–3 show that these two algorithms effectively solve the benchmark problems—except for the fourth problem. In the experiment, the results when the algorithms are applied to problems 2 and 3 are not satisfactory when the dimensions of the problem are large; thus, we use a lower dimension. The dimensions of problems 8 and 9 are less than 1,000; the reasoning is similar to that for problems 2 and 3. However, the dimensions of all the problems are larger than 30, which is fixed. For many problems, the results are similar, except for those of problem 6. Clearly, the restart algorithm is competitive with the normal algorithm without using the restart technique.

To clearly to show the performance of Algorithm 4.1 and Algorithm N on NFG, we use the tool (S1 File) in [46] to analyze the algorithms. The results are listed in Table 4. It is easy to see that Algorithm 4.1 outperforms Algorithm N by approximately 1%, and we can conclude that the proposed method is better than the normal method. Thus, we hope that the given algorithm will be utilized in the future.

**Table 4:** The performance of Algorithm 4.1 and Algorithm N on NFG.

Algorithm 4.1	Algorithm N
0.99	1

### Conclusion

1. This paper presented a new conjugate gradient method that exhibits global convergence, linear convergence, and quadratic convergence when suitable assumptions are made. Our proposed CG formula includes not only the gradient value information but also the function value information. For the test benchmark problems, the numerical results showed that the given algorithm is more effective than the normal method without using the restart technique.
2. We performed tests using benchmark problems using the presented algorithm and the normal algorithm without employing the restart technique. These two methods were shown to be very effective for solving the given problems. Moreover, we did not fix the dimension because *n* = = 30, and the largest dimension was higher than 100,000 (103,000). Additional numerical problems (such as the problems in [47]) should be investigated in the future to examine this algorithm.
3. Recently, we solved nonsmooth optimization problems using the relative gradient methods and obtained various results; therefore, in the future, we will use the RMPRP* method to solve nonsmooth optimization problems and hopefully obtain interesting results. Moreover, we will study the convergence of the CG method with other line search rules.

## Supporting Information

S1 File Supporting Information for Table 4. (PDF)
