# Supplementary material for: A Conjugate Gradient Algorithm with Function Value Information and N-Step Quadratic Convergence for Unconstrained Optimization
Source: PLoS One. 2015 Sep 18;10(9):e0137166. doi: 10.1371/journal.pone.0137166 (PMC4575111; doi:10.1371/journal.pone.0137166)
Supplement: S1 File — (PDF) [file pone.0137166.s001.pdf]

Supporting Information Captions

S1 File. Supporting Information for Table 4. In this part, we use the Empirical analysis [47] to measure the performance of an algorithm. In order to rank the iterative numerical methods, we compute the total number of function and gradient evaluations by the formula

$$N_{total} = NF + mNG$$

where NF and NG denote the number of function evaluations and gradient evaluations respectively, and m is a positive integer. In this paper, we take $m = 1$.

Table 2 and 3 have presented the N_{total} for each test function and we denote them by $N_{total,i}(Algorithm4.1)$ and$N_{total,i}(AlgorithmN)$, then calculate the ratio,

$$r_i(Algorithm4.1) = \frac{N_{total,i}(Algorithm4.1)}{N_{total,i}(AlgorithmN)}$$

If the Algorithm 4.1 does not work for problem i_0, we replace the $r_{i_0}(Algorithm4.1)$ by a positive constant κ which is defined as follows:

$$\kappa = \max\{r_i(Algorithm4.1)) : (i, j) \notin S_1\}$$

where

$$S_1 = \{i : Algorithm4.1 \;\; does\ not\ work\ for\ problem\ i\}.$$

The geometric mean of these ratios for Algorithm 4.1 over all the test problems is defined by

$$r(Algorithm4.1)) = [\prod_{i \in S_1} r_i(Algorithm4.1)]^{1/|T|}$$

where T denotes the set of the test problems and $|T|$ is the number of elements in T. The comparison is relative and hence does not be dominated by a few problems for which the method requires a great deal of function evaluations and gradient functions.

According to the above rule, it is clear that r(Algorithm N) = 1. The values of r(Algorithm 4.1)is listed in Table 4.
